# Propagation of cell death in *dropdead1*, a sorghum ortholog of the maize *lls1* mutant

**DOI:** 10.1371/journal.pone.0201359

**Published:** 2018-09-10

**Authors:** Anoop Sindhu, Diane Janick-Buckner, Brent Buckner, John Gray, Usha Zehr, Brian P. Dilkes, Gurmukh S. Johal

**Affiliations:** 1 Department of Botany and Plant Pathology, Purdue University, West Lafayette, Indiana, United States of America; 2 Department of Biology, Truman State University, Kirksville, Missouri, United States of America; 3 Biological Sciences Department, University of Toledo, Toledo, Ohio, United States of America; 4 Department of Agronomy, Purdue University, West Lafayette, Indiana, United States of America; 5 Department of Biochemistry, Purdue University, West Lafayette, Indiana, United States of America; 6 Center for Plant Biology, Purdue University, West Lafayette, Indiana, United States of America; University of Maryland Baltimore County, UNITED STATES

## Abstract

We describe *dropdead1-1* (*ded1*), an EMS-induced recessive lesion mimic mutant of sorghum. It is characterized by the formation of spreading necrotic lesions that share many attributes with those associated with the maize *lethal leaf spot1* (*lls1)* and Arabidopsis *accelerated cell death1* (*acd1*) mutation. We show that as in *lls1*, *ded1* lesions are initiated by wounding and require light for continued propagation, and that loss of chloroplast integrity is responsible for *ded1* cell death. Consistent with these parallels, we demonstrate that *ded1* is an ortholog of *lls1* and encodes pheophorbide *a* oxidase (PaO) with 93% identity at the protein level. The mutant *ded1* allele resulted from a stop codon-inducing single base pair change in exon 6 of the sorghum ortholog of *lls1*. The *ded1* transcript was rapidly and transiently induced after wounding and substantially elevated in leaves containing *ded1* lesions. Given that PaO is a key enzyme of the chlorophyll degradation pathway, its dysfunction would result in the accumulation of pheophorbide, a potent photosensitizer that results in the production of singlet oxygen. Consistent with this, cell death associated with *ded1* lesions is most likely caused by singlet oxygen as our results exclude superoxide and H_2_O_2_ from this role. We explore the signal responsible for the propagation of lesions affecting both *ded1* and *lls1* lesions and find that both developmental age and ethylene increase the rate of lesion expansion in both mutants.

## Introduction

A number of plant mutants undergo spontaneous cell death in the absence of any obvious stress or injury (reviewed in [1,2]). As such lesions often resemble those produced during plant interactions with pathogens, these mutants may be referred to as “disease lesions mimics” or simply “lesion mimics” [1,2]. A subset of lesion mimic mutants do encode disease signaling components resulting in constitutive hypersensitive-response [1,2]. Dominant lesion mimic mutants typically encode constitutively active alleles of genes that activate cell death. Recessive lesion mimics may encode negative regulators of cell death signaling. Recessive mutants may also encode alleles of host components (guardees) that are guarded by host immune receptors (guards) if the guardee is directly or indirectly targeted by pathogen effectors [1]. But not all lesion mimics are mutations in disease signaling components. Lesion mimics have been isolated that encode enzymes that when disrupted result in the accumulation of biochemical intermediates, including phototoxic compounds from chlorophyll metabolism. The first lesion mimic mutant was described in maize by Emerson in 1923 who described the phenotype as “blotched leaf” [3]. The observation that these maize mutants phenocopy disease symptoms was made later, by Neuffer in the 1970s [4]. Disease lesion mimics have been found in many plant species including *Arabidopsis*, barley and rice, where they were variously named as *accelerated cell death*, *lesions simulating disease* or *spotted* mutants [5–7]. More than 50 lesion mimic mutants have been described in maize and a similar number of mutants are present in *Arabidopsis* [1,8,9].

The lesions on lesion mimic mutants can be determinative lesions, that reach a defined maximum size, or produce propagative lesions that expand until they engulf the entire organ [1]. In the determinative class, lesions initiate often profusely but then remain restricted in size, resembling a massive number of hypersensitive cell death (HR) sites. It is presumed that lesions in the determinative class result from impairments that either lower the threshold for cell death initiation or cause the buildup of factors or metabolites to cytotoxic levels. In contrast, propagative mimics are thought to result from defects in those genes that negatively regulate cell death in plants [1]. The ubiquitous feature of lesion mimics is their association with aberrant cell death. Thus, this class of mutants should identify genes and mechanisms that control programmed cell death (PCD) [1,2].

PCD in plants plays a key role in development and the defense of plants against biotic and abiotic stresses [1,10–12]. However, our knowledge of how plants accomplish PCD remains limited. In contrast, great advances have been made in animals demonstrating the cellular machinery of a major PCD pathway, termed apoptosis, is largely conserved from worms to humans [13–19]. No such framework of genes and mechanisms has yet emerged for the control and execution of PCD in plants, although several proteases have been identified as having a role in plant PCD [20]. Triggering of PCD by defects in signaling for disease resistance results reactive oxygen species (ROS) production and constitutive expression of defense genes [21]. ROS production is a normal component of the disease resistance signaling and adaptive response to pathogens. As a result, many mutants with altered disease resistance also affect aberrant PCD or ROS production [22–24]. This inappropriate production of reaction oxygen species is a feature that is shared across all lesions mimics, whether arising from errors in metabolism or defects in defense signaling. The exploration of lesion mimic phenotypes has assisted in identifying varied mechanisms capable of inducing PCD in plants as genes underlying a number of these mutants have been cloned and characterized [9,25]. In addition to disease signaling and metabolic defects, transcriptional sensors of ROS levels have also been identified as capable of producing lesion mimic mutants. One example is the mutant *LSD1*, initially thought to result in cell death because of disruption of ROS associated homeostasis [6,26,27]. However, a recent study has shown that cell death associated with *lsd1* is suppressed by mutations in an NLR [28].

Approximately 50% of the lesion mimic mutations result from mutations in genes not involved in disease resistance. A good proportion of these are the result of errors in metabolism, especially due to the accumulation of phototoxic intermediates. Two examples involve mutations that either block the production of chlorophyll or those that block the degradation of chlorophyll. Loss of function at *acd2* in Arabidopsis blocks chlorophyll degradation and results in a lesion phenotype from the accumulation of a phototoxic catabolic intermediate that produces singlet oxygen in the light [29]. As an indication of the complexity in interpreting these mutants, overexpression of *acd2* was shown to suppress cell death and disease symptom expression in an incompatible pseudomonas interaction with Arabidopsis, yet this effect of ACD2 overexpression was not dependent on chlorophyll [30]. A similar lesion phenotype is affected by mutations in *lls1* in maize and *acd1* in Arabidopsis. These genes encode the pheophorbide *a* oxidase (PaO) activity, one step upstream of *acd2* in chlorophyll catabolism, and blocking these genes accumulates a phototoxic catabolic intermediate that also produces singlet oxygen in the light [31,32]. Thus, lesions from ROS production via singlet-oxygen can be powered by aberrant light capture.

These studies highlight the complexity of mechanisms regulating cell death in plants and it remains largely unaddressed how cell death associated with most lesion mimics is initiated and propagated. One exception in this regard is the afformentioned recessive mutant of maize, *lls1* [33]. Initiation of *lls1* lesions is triggered in response to cell damage, and propagation of these lesions by continued enlargement is fuelled by chemical conversion of photosynthetically-active light into singlet oxygen [34]. The PAO enzyme encoded by *lls1* catalyzes the cleavage of the pheophorbide a macrocycle yielding the open-tetrapyrrolic backbone structure of different types of Chl catabolites found in senescent leaves and fruits [35,36]. The formation of lesions in plants lacking PaO is conserved across plants, as expected for a metabolic defect that induces ROS and cell death. Mutants in PaO in multiple plant species including the Arabidopsis *accelerated cell death 1* (*acd1)* mutant, rice *early senescence1* (*eas1*) mutant, VIGS of PaO in tomato, and knockdown of all the wheat PaO orthologs results in lesion formation [5,37–41].

The continued enlargement of lesions in propagative mutants, such as *lls1*, requires a diffusible signal. The nature of this signal in *lls1* is unknown, but chloroplasts play a central role in the propagated cell-autonomous cell death in *lls1* lesions [33,34]. Extensive work in Arabidopsis has pointed to the existence of multiple factors that promote or suppress cell death in response to singlet oxygen [42–45], the ROS that results from photoactivation of pheophorbide A [44,46]. Multiple studies of singlet oxygen-induced lesions have identified pathways that modulate lesion formation in response to singlet oxygen including salicylic acid and ethylene signaling [46,47]. This suggests that modulation of hormone levels, or perception, might enhance or suppress cell death in mutants that accumulate singlet-oxygen evolving metabolic intermediates. Singlet oxygen production in the chloroplast is a feature, particularly in high light, of the light harvesting process and not just an aberrant consequence of metabolic defects. Multiple chloroplast components play roles in quenching singlet oxygen, including carotenoids and tocopherols. A number of mutants have defined proteins of unknown function as modulators of singlet oxygen-induced lesions, including *executer1* and *executer2*, and *flu* [42,48]. The existence of such proteins, and the modulation of singlet oxygen lesions by a wide variety of biochemical and physiological pathways, raises the possibility that singlet oxygen production could be a component of a disease response as either a mechanism to produce cytotoxic ROS during adaptive disease resistance or as a mechanism to induce cell death for nectrotropic growth of pathogens.

The focus of this paper is an EMS-induced recessive lesion mimic mutation from sorghum that we name *dropdead1* (*ded1*). Many features of this mutation were strikingly similar to that of *lls1*. Here we demonstrate that the *ded1* mutant results from a single base pair change in a sorghum ortholog of the maize *lls1* gene. Like the *lls1* mutant, *ded1* lesions are mediated by a light-dependent mechanism that disrupts the integrity of chloroplasts to kill *ded1* cells. Neither superoxide nor H_2_O_2_ were detectably elevated and appear not to be involved in this cell death which, given the PAO deficiency caused by *ded1-1* and *lls1-1* mutations, is likely mediated by singlet oxygen.

## Results

### Phenotypic characterization of *ded1*

**Fig 1:**
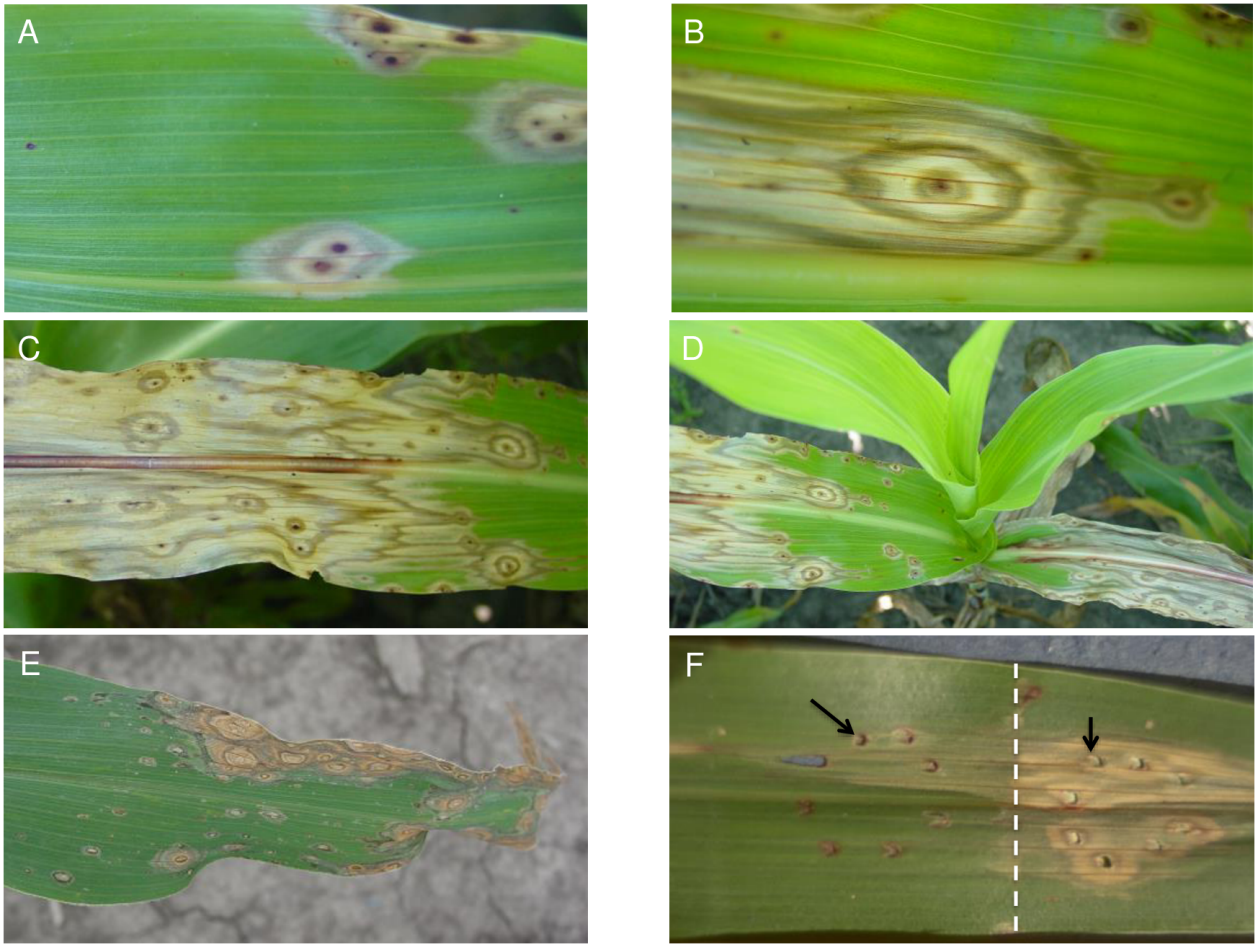
Phenotypic manifestation of *ded1*. **(A, B)** Typical morphology of *ded1* lesions forming spontaneously on leaves of field-grown mutants. Note the bull’s eye appearance of the lesions. **(C)** Typical blighting of a *ded1* leaf as lesions spread and merge with each other **(D)** A field grown *ded1* plant exhibiting developmental progression of the mutant phenotype **(E)** Maize *lls1* lesions for comparison with ded1 lesions **(F)** Leaf of a *ded1* plant subjected to 8 pin-prick wounds (black arrows) in two regions, one was covered with aluminum foil and one was exposed to light. Note the lack of development of lesions on the region of the leaf that had been covered with the foil (border indicated by white dashed line).

The *ded1* is a recessive mutation that originated in an EMS mutagenized M2 population of P898012 developed at Purdue University. The *ded1* mutants form necrotic lesions that expand and coalesce, ultimately consuming whole leaves (Fig 1A–1D). Lesion formation is under some developmental control and initiate on mature leaves (Fig 1D). Repeated observation of successive generations of mutant families in more than five field seasons indicated that both phenotype expression and the developmental control of lesion initiation were stable and reproducible. The same phenotypes were observed in greenhouse-grown *ded1-1* mutants. Lesions form on the first leaf of greenhouse-grown plants two to three weeks after emergence. Lesions initiate at or near the tip of the first leaf and progress toward the developmentally younger tissue at the base of the leaf. Over a few days, the area containing dead and dying cells increases until independently initiated lesion sectors merge with each other. This pattern of lesion formation and spread is repeated on all successive leaves and completely blights all of the foliage shortly after flowering. *ded1* lesions often accumulate red pigments (presumably anthocyanins and phlobaphenes) in the center of the lesion and to a lesser extent in concentric rings, giving the lesions a characteristic “bull’s eye” appearance (Fig 1A and 1B).

Injuring the mutant leaf tissue can trigger lesion formation in *ded1-1* mutants. Mechanical penetration of leaf tissue of sufficient developmental age resulted in the rapid formation of lesions (Fig 1F). Wounding of younger leaf tissue resulted in wound sites that remained unaffected so long as the leaf was immature. However, when the leaf bearing the wound aged, competency to bear lesions developed in the injured leaf and some of the wound punctures gave rise to typical expanding *ded1* lesions.

The continued expansion of *ded1* lesions requires light. Covering wounded portions of mutant leaves with aluminum foil prevented the formation of *ded1* lesions (Fig 1F). This phenomena was also true for preexisting lesions undergoing expansion, which could be halted by protecting leaves from light (data not shown), clearly demonstrating the importance of light in *ded1* lesion ontogeny.

### *ded1* lesion growth is associated with structural disruption of chloroplasts

**Fig 2:**
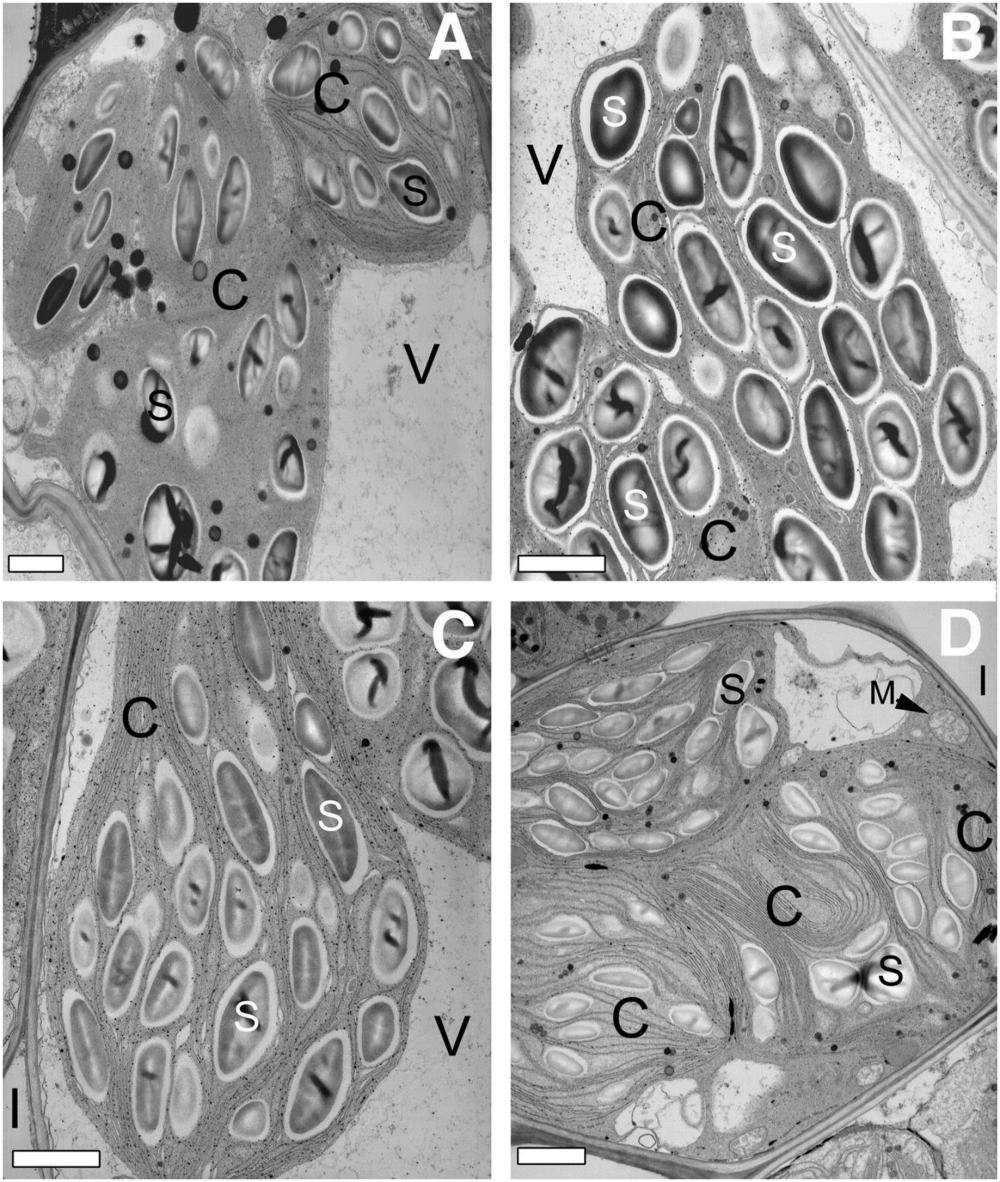
Transmission electron microscopy of bundle sheath cells in uninjured and injured (21 hours post wounding) wild-type and *ded1* leaves. **(A)** Bundle sheath cell in uninjured wild-type leaf tissue. **(B)** Bundle sheath cell adjoining dead cells in injured wild-type leaf tissue. **(C)** Bundle sheath cell in uninjured *ded1* leaf tissue. **(D)** Bundle sheath cell adjoining dead cell in injured *ded1* leaf tissue. Note the rounded appearance of the chloroplasts and folding of the thylakoid membranes. Bars = 1 μm. V = vacuole; S = starch; C = chloroplast; I = intercellular space; M = mitochondrion.

**Fig 3:**
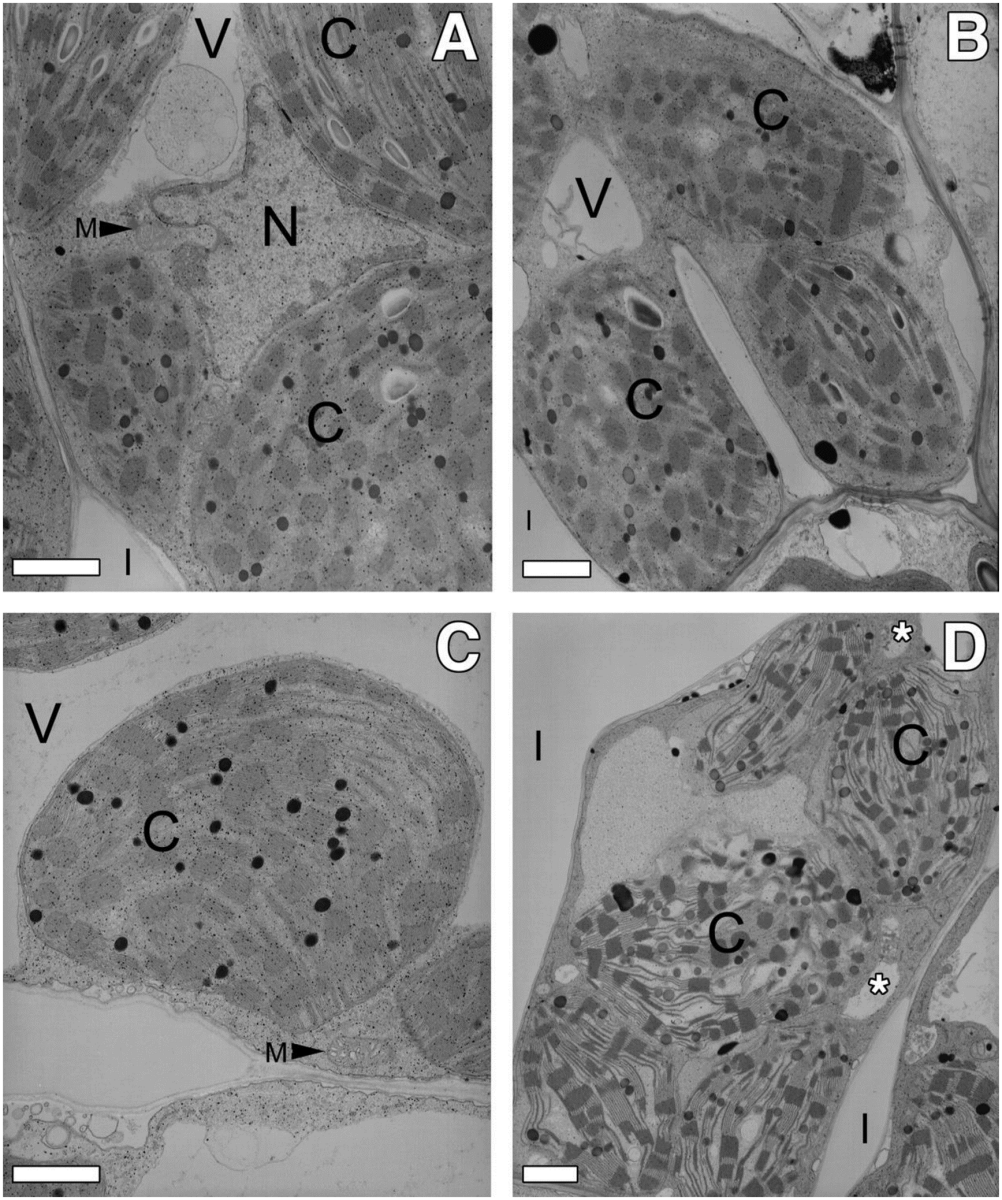
Transmission electron microscopy of mesophyll cells in uninjured and injured (21 hours post wounding) wild-type and *ded1* leaves. **(A)** Mesophyll cell in uninjured wild-type leaf tissue. **(B)** Mesophyll cell adjoining dead cells in injured wild-type leaf tissue. **(C)** Mesophyll cell in uninjured *ded1* leaf tissue. **(D)** Mesophyll cell adjoining dead cell in injured *ded1* leaf tissue. Note the swelling of the chloroplasts. Asterisks indicate location of cytoplasmic vacuoles. Bars = 1 μm. V = vacuole; C = chloroplast; I = intercellular space; M = mitochondrion; N = nucleus.

The visible phenotype of *ded1* lesions, their initiation in response to physical injury, and dependence on light are similar to the maize *lls1* mutant. *lls1* lesions are characterized by swelling and disruption of chloroplasts [34]. To further explore the similarity of *ded1* and *lls1*, we used transmission electron microscopy to examine the chloroplast ultrastructure during lesion formation in *ded1* cells. Pin-prick wounds were made on wildtype and *ded1* mutants to initiate lesions. Cells surrounding pin-prick wounds were examined and compared to uninjured tissue collected from an equivalent area of the same *ded1* or wild-type leaf on the opposite side of the mid-rib. Cells were examined at 21h and 42h after wounding, and images collected from 21h tissue are shown (Figs 2 and 3). Although the lesions had progressed farther from the wound site in the 42h samples, identical ultrastructural changes were found at both 21h and 42h after wounding. Like *lls1* mutants, *ded1* cells from uninjured tissue were indistinguishable from wild-type cells (Figs 2a, 2c, 3a and 3c). As with *lls1* cells, cells near pin-prick damaged *ded1*, but not wildtype or uninjured *ded1* cells, exhibited dramatic changes in chloroplasts of both bundle sheath (BS) and mesophyll (M) cells (Figs 2 and 3). Chloroplasts of BS cells adjacent to the wound looked disorganized and convoluted and their thylakoid membranes appeared to have folded over upon themselves (Fig 2d). Chloroplasts in M cells adjacent to wounded *ded1* cells were swollen (Fig 3d). The thylakoid membrane stacks were somewhat pulled apart in these swollen chloroplasts, but maintained a normal organization (Fig 3d). The envelope of these swollen chloroplasts appeared to be intact and no ruptured M chloroplasts were observed. Just as in *lls1* mutants, the severity of chloroplast ultrastructural changes in BS and M cells decreased with the distance from the injured *ded1* cells (data not shown). Taken together, these data suggest that, as in the *lls1* mutation, the changes that result in cell death during propagation of lesions in *ded1* plants are initiated in chloroplasts.

In addition to the chloroplast changes, M and BS cells of wounded *ded1* plants displayed vacuolization of their cytoplasm (Figs 2d and 3d). Though their presence was clear and reproducible, the origin of these vacuoles was not clear. However, the central vacuole was conspicuously absent in all M and BS cells that exhibited severely altered chloroplast morphology, suggesting a loss of tonoplast integrity. Cytoplasmic vacuolization was never seen in the absence of chloroplast structural changes. Mitochondria, Golgi and endoplasmic reticuli (ER) in BS or M cells of injured *ded1* tissue were comparatively normal, even in cells displaying dramatically altered chloroplast structure (Figs 2d and 3d). Collapsed M and BS cell corpses appeared condensed and shrunken (data not shown).

Not all of the ultrastructural changes in dying *ded1* were similar to *lls1*. The nuclei of wounded *lls1* cells displayed increased heterochromatin located at the nuclear envelope [34]. No such increase in heterochromatin was observed in the nuclei of dying *ded1* M and BS cells. Thus this morphological change, which occurs in apoptotic cells [13], is not necessary for cell death associated with *ded1* lesions.

### The *ded1* gene encodes the sorghum ortholog of *lls1*

The similarity between the *ded1* and *lls1* phenotypes suggested that they may result from mutations in the same gene. The sorghum ortholog of *lls1*, Sobic.001G504900 resides in a syntenic position and shows one-to-one orthology with the maize *lls1* gene. Sobic.001G504900 was amplified and sequenced to see how it compared between the *ded1* mutant and its wild-type progenitor. A PCR fragment from exon 5 through exon 7 of approximately 1.1 kb was obtained from both the *ded1* mutant and progenitor DNAs. These products, designated dd57-11M and dd57-11P for mutant and progenitor amplicons, respectively, were cloned and sequenced. The *ded1* mutant encodes a transversion of C to A at the 77,311,393 nucleotide position of chromosome 1 in sorghum genome (version 3.1.1), within the coding sequence of Sobic.001G504900. This substitution causes a nonsense mutation that converts the Serine encoding TCA codon at the 437 amino acid position of the Sobic.001G504900.1.p protein model to an ochre stop codon. Thus, Sobic.001G504900 in *ded1-1* now encodes a truncated, and presumably non-functional, DED1 protein.

**Fig 4:**
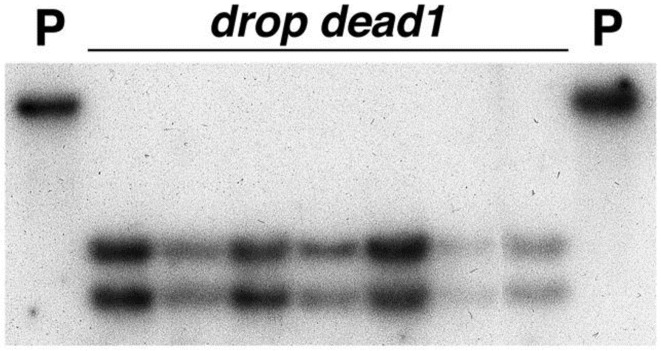
DNA blot hybridization analysis demonstrating linkage of the *ded1* phenotype with a restriction fragment length polymorphism generated from the single base pair change in *ded1* allele. 10 μg of DNA pooled from 5 *ded1* plants (labeled “*dropdead1*”) and 2 or 3 wild-type (labeled “P”) plants homozygous for the progenitor allele was digested with *DraI* and *EcoRI* restriction endonucleases, run on a gel, blotted and hybridized with ^32^P-labeled dd57-11M.

This mutation created a *DraI* cleavage site in the *ded1-1* allele that was absent in its progenitor’s sequence. DNA from a total of 45 *ded1* plants and 5 wild-type plants homozygous for the progenitor allele was isolated and digested with *DraI* and *EcoRI* restriction endonucleases. DNA blot hybridization analysis of these DNAs using dd57-11M as a hybridization probe demonstrated that the *DraI*/*EcoRI* digested DNA from wild-type plants possessed a single band of approximately 4.4-kb (Fig 4). Two bands (approximately 2.7-kb and 1.7-kb) were present in lanes containing *DraI*/*EcoRI* digested DNA isolated from *ded1* plants (Fig 4). All *ded1* mutants analyzed in this study were found to possess the *DraI* polymorphism indicating perfect co-segregation of this polymorphism and the mutant phenotype in these 45 mutants. Cosegregation was confirmed using flanking markers genotyped by Restriction Fragment Length Polymorpism (RFLP) scoring of southern blots (data not shown). These confirmed the position of DED1 and placed RFLP markers *PIO000689* and *PIO000603* mapped 1cM and 7cM distal to *ded1* and *UMC157* 7cM proximal to *ded1*. Another marker *PIO000640* was found to reside 18 cM distal to *ded1*.

An additional F_2_ population was generated between *ded1* and the Shanqui Red inbred line. Fifty plants from this F_2_ population, 45 mutant and 5 wild-type, were genotyped for these RFLP. The RFLP marker *UMC 157* was found to map about 4 cM away from the *ded1* locus with this population. Whether it located proximally or distally in relation to *ded1* could not be established because both *PIO000603* and *PIO000689* segregated completely with the *ded1* locus. Even *PIO000640*, which mapped 18 cM distal to *lls1* in maize, failed to recombine with the *ded1* gene in this population (data not shown). As expected, the *DraI* polymorphism within Sobic.001G504900 co-segregated perfectly with the mutant phenotype. These mapping data, and the presence of a nonsense mutation within Sobic.001G504900 demonstrate that *ded1* is encoded by the ortholog of the maize *lls1* gene.

### The *ded1* gene is rapidly induced by wounding in a transient fashion

**Fig 5:**
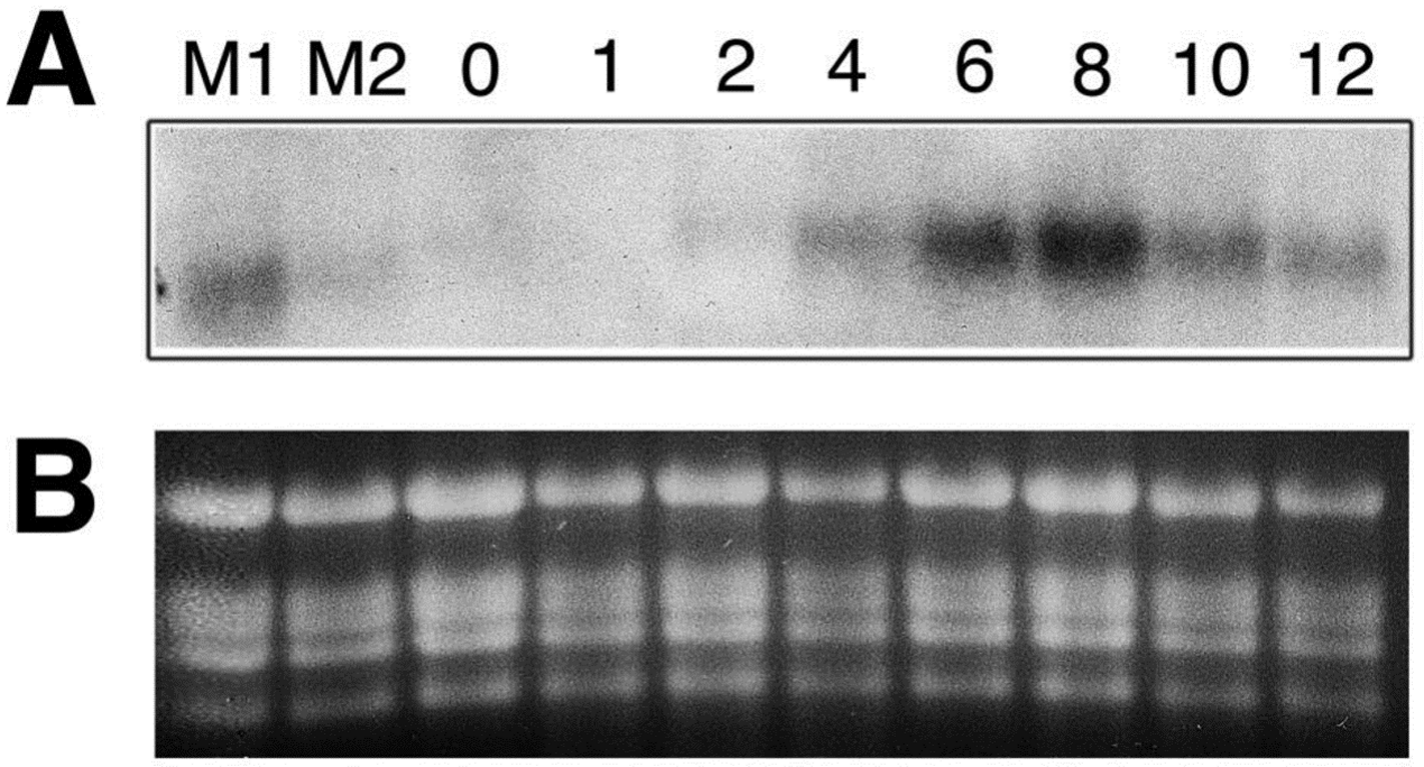
RNA blot hybridization analysis of RNAs isolated from *ded1* and wild-type sorghum leaves. Wild-type leaves were wounded by gently rubbing carborundum powder down the entire length of the leaf. Total RNA was isolated from tissue collected at 1, 2, 4, 6, 8, 10 and 12h post wounding, as well as prior to wounding (0 hours). **(A)** 10 μg total RNA was loaded per lane, size fractionated by electrophoresis, blotted and hybridized with the ^32^P-labeled dd57-11M fragment. RNA in lane M2 was isolated from a leaf of a *ded1* plant with a low number of lesions, whereas the RNA in Lane M1 was isolated from a *ded1* plant leaf with a moderate number of lesions and contains a higher *lls1* signal. **(B)** Ethidium bromide stained gel showing the rRNA bands was used as a loading control.

RNA gel blot analyses of DED1 transcript accumulation identified no change in transcript size, supporting the single nucleotide substitution origin of the *ded1-1* allele. However, the accumulation of DED1 transcript in *ded1-1* was elevated many fold, indicating a lack of nonsense-mediated decay of this allele’s gene product. The level of DED1 transcript appeared to roughly predict the number of lesions on the leaf from which the RNA was extracted (Fig 5 lanes M1 and M2). Mutant leaves that had a higher number of lesions (lane M1) had a larger increase in the level of the *ded1* transcript than the mutant leaves that had a relatively fewer number of *ded1* lesions (lane M2). This suggested that DED1 was induced during lesion formation and that, like the *ded1* lesions, the *ded1* gene expression was responsive to tissue damage resulting from spreading lesions. To investigate this possibility further, leaves of 5 week-old plants were gently rubbed with carborundum powder [49]. This maximized the number of cells experiencing the wounding stimulus. Total RNA was isolated at 1, 2, 4, 6, 8, 10 and 12h after wounding and subjected to RNA blot hybridization analysis using dd57-11 as a probe. As shown in Fig 5, a rapid and transient induction of DED1 mRNA was observed in response to wounding. The DED1 transcript started accumulating around 4h after wounding and reached the peak at 8h. This peak was followed by a sharp decline and return to the amount of the DED1 transcript detected at 4h by 12h after wounding (Fig 5).

### Ethylene but neither H_2_O_2_ nor superoxides were associated with the propagation of cell death in *ded1-1*

**Fig 6:**
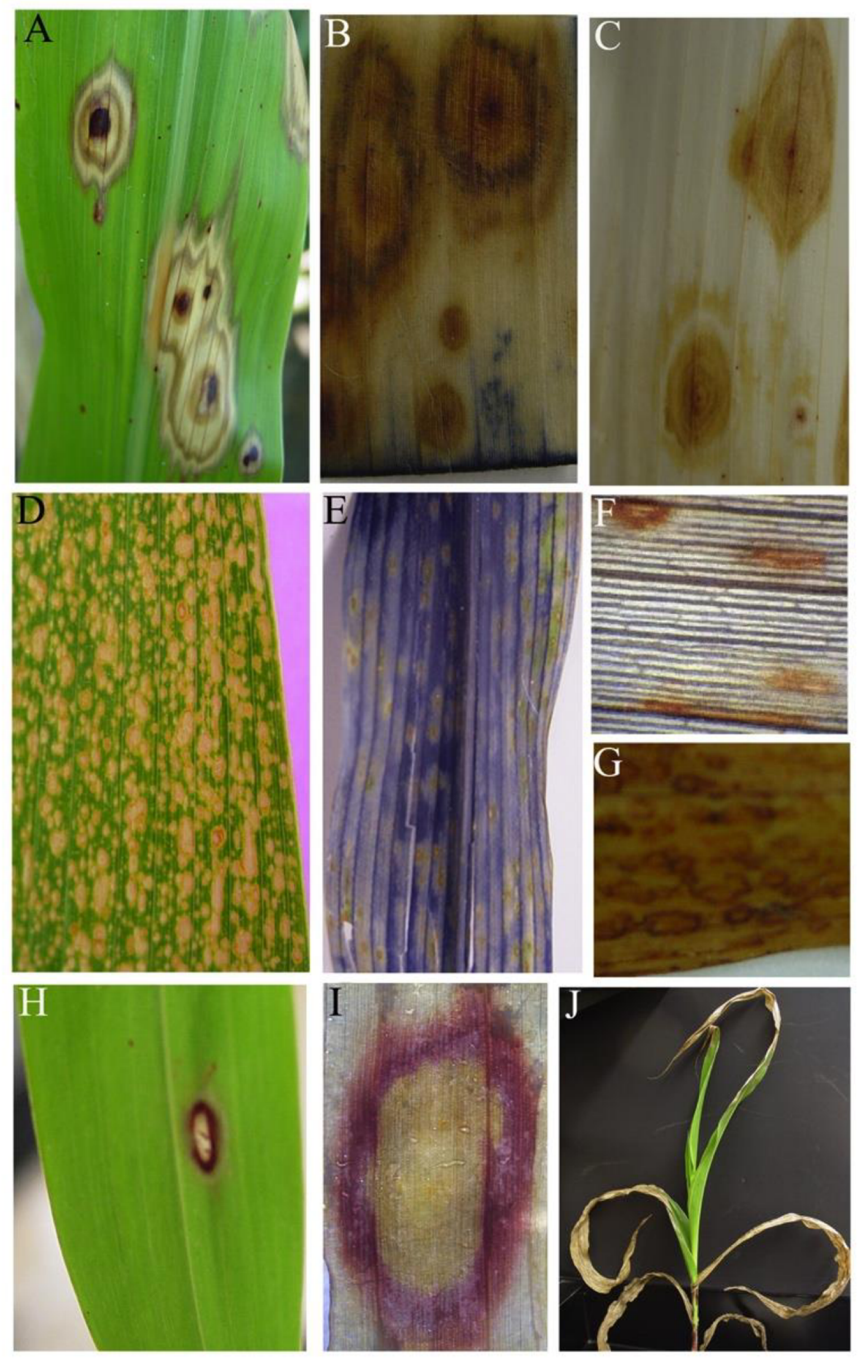
Induction and propagation of cell death associated with *ded1* lesions. **(A)** Actively expanding *ded1* lesions. **(B,C)** Lack of superoxide and H_2_O_2_ production in and around *ded1* lesions. Tissue shown in b was examined for superoxide using the NBT staining assay. Lesions in c were stained with DAB to detect H_2_O_2_. **(D)** A maize leaf exhibiting Les*-101 lesions. **(E)** Positive staining of a Les*-101 leaf with NBT, indicating the presence of superoxide. **(F,G)** Positive staining of Les*-101 lesions with DAB, indicating the presence of H_2_O_2_. **(H)** A ring of red pigments that forms at the periphery of *ded1* lesions following protection from light for at least 48h. **(I)** A close up (following clearing of chlorophyll) of the red ring circling a *ded1* lesion. **(J)** Rapid collapse of mature leaves of *ded1* plants following spray with ethephon, an ethylene releasing chemical.

The dependence of *ded1* lesion formation on light and the role of the *ded1* gene product in chlorophyll catabolism suggests that reactive oxygen species may be involved in the formation of lesions. To address this, we assayed the production superoxide and H_2_O_2_ in actively expanding lesions on *ded1-1* leaves using NBT and DAB staining (Fig 6). These histochemical assays provide in-vivo and in-situ visualization of respective oxygen free radicals with high sensitivity [50]. The Les*-101 maize mutant was used as a positive control. Presence of O_2_^-^ is indicated by the blue coloration and presence of H_2_O_2_ is indicated by reddish-brown coloration, as shown in the lesions of Les*-101 mutant maize leaf lesions (Fig 6D–6H). Cell death in expanding *ded1* lesions occurs at the boundary, if ROS production causes cell death we expect a positive stain reaction only at the lesion periphery similar to the red ring of anthocyanins/phlobaphenes around *ded1* lesions that are protected from light (Fig 6H and 6I). No positive reaction was observed with either NBT or DAB assays in *ded1-1* leaves (Fig 6B and 6C), demonstrating a lack of superoxide and H_2_O_2_ involvement in *ded1* lesion ontogeny.

The requirement for wounding raised the question of what aspect of wound signaling could mediate lesion expansion in *ded1-1* mutants. Previous work demonstrated that the *acd1* mutant was dramatically enhanced by the application of ethylene [5]. We utilized the commercial plant growth regulator ethephon 0.01% as an aqueous spray to increase ethylene levels in planta. Ethephon is efficiently converted from a stable form into the plant hormone ethylene by cellular metabolism. Just as was the case for *acd1* mutants [5] the *ded1-1* mutant was profoundly sensitive to ethephon resulting in collapse of all sprayed tissue within days of application (Fig 6J). As this had not been investigated in the *lls1* mutant we tested application of ethephon to *lls1* and found similar dramatic induction of lesions following 0.01% ethephon spray (data not shown). The extent of lesion enhancement resulted in complete consumption of all sprayed leaves in all plants in both the *ded1-1* sorghum and *lls1* maize mutants. Remarkably, ethephon application induced lesions and resulted in complete collapse of all leaves in plants as young as 3 weeks-post-sowing (Fig 6J), suggesting that ethylene was sufficient to induce chlorophyll degradation and expose the defect in *ded1-1*.

### The *ded1* gene is developmentally induced in adult leaves, during pathogen stress and during osmotic stress

**Fig 7:**
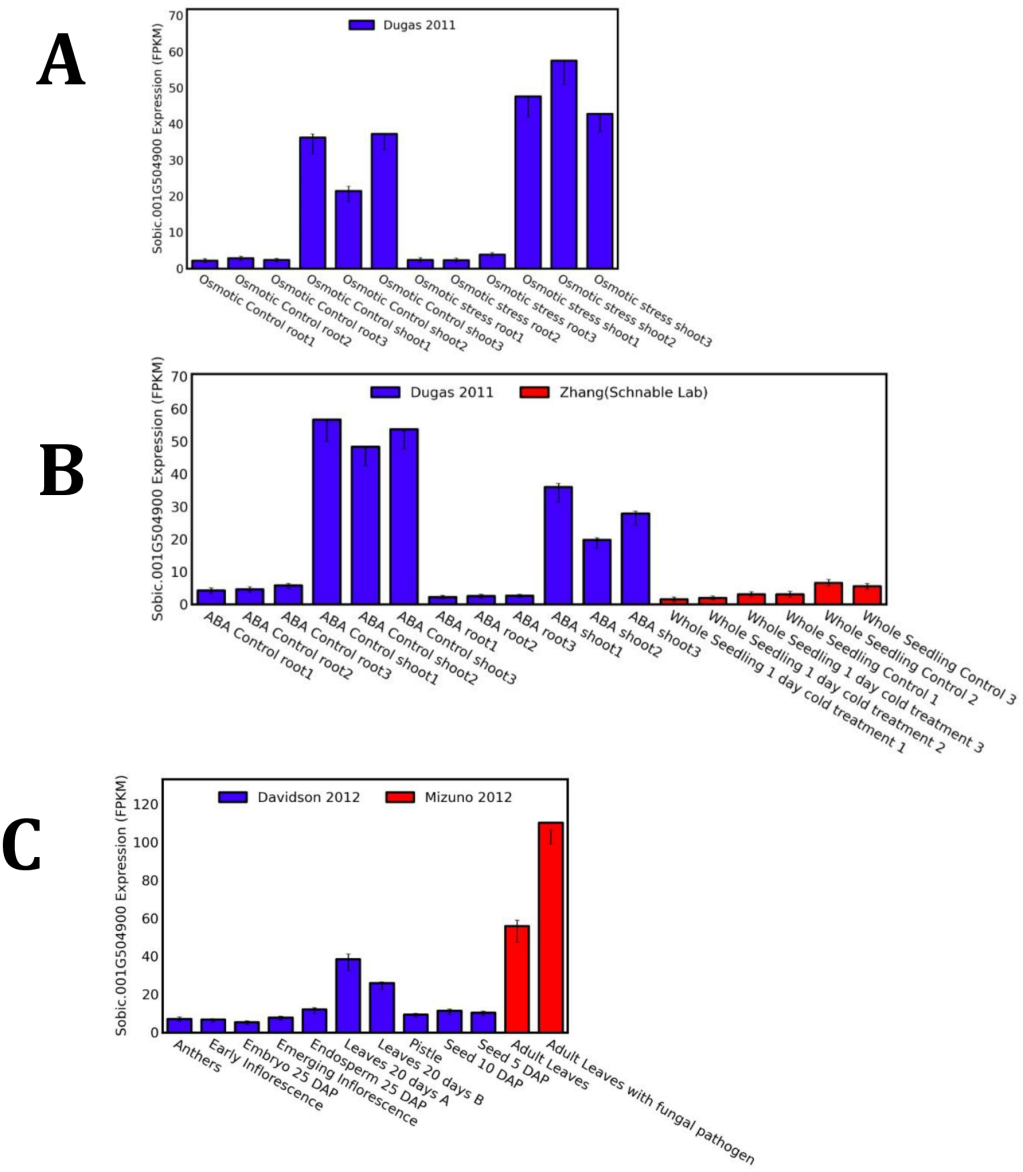
Transcript profiling of the DED1 gene product during development, pathogen stress, osmotic stress, and ABA application. **(A)** Developmental variation in DED1 transcript accumulation (Data source—Davidson et al 2012) and in response to infection by *Bipolaris sorghicola* (Data source -Mizuno *et al*., 2012). **(B)** Expression of the DED1 transcript in seedling shoots and roots in response to osmotic stress (Data source Dugas *et al*., 2011). **(C)** Relative accumulation of DED1 gene product in response to ABA treatment (Data source Dugas *et al*., 2011) or cold stress (Data source—Zhang and Schnable Lab BioProject PRJNA343268). All RNAseq datasets are normalized as fragments per kilobase of exon per million fragments mapped (FPKM) using qTeller (http://qteller.com).

The induced expression of the *Ded1* gene during wounding and ethephon treatment prompted us to survey the expression of this gene in existing RNA-seq datasets available through the qTeller database (www.qteller.com). The *Ded1* transcript, normalized to facilitate comparative expression profiling, was compared across a variety of developmental stages and in response to externally applied stresses. The survey reveals that the *Ded1* gene is expressed at relatively low levels in tissues other than leaves, transcript accumulation increased as leaves matured, and reached a high level in adult leaves (Fig 7A). When mature leaves were challenged by inoculation with conidia of *Bipolaris sorghicola*, the *Ded1* transcript increased two fold (Fig 7A). The expression of the DED1 transcript increased by about 20% following osmotic stress in shoots but no detectable increase was observed in the roots (Fig 7B). One day of cold-stress had no effect on DED1 expression in whole seedlings (Fig 7C). Lastly, the application of ABA to seedling shoots and roots resulted in a ~40% reduction in DED1 transcript accumulation.

## Discussion

To our knowledge, *ded1* is the first lesion mimic mutation to be molecularly identified in sorghum. It is a recessive mutation of the propagative class in which lesions, once formed, expand continuously to engulf the entire leaf. It was isolated from an EMS-mutagenized M2 population by late Dr. John Axtell’s group in the Department of Agronomy at Purdue University. It was also Dr. Axtell who, after having attended a seminar on the phenotypic behavior of *lls1*, first predicted a relationship between these two mutants. We now establish this connection by showing that *ded1-1* resulted from a single base pair change in the sorghum ortholog of the maize *lls1* gene. The mutagenesis resulted in a C to A transversion, not typical of EMS mutagenesis, and it induced a premature stop codon that truncated the protein.

This mutation did not change the size of the DED1 transcript but DED1 transcript accumulation increased in *ded1* leaves with lesions. This revealed a feature of the *ded1* gene that relates to its induction in response to cellular damage. As shown here and also for *lls1* [33,34], *ded1/lls1* undergoes rapid transcription but transient induction in response to cellular damage including physical injury of the tissue. This feature of the *ded1* gene, along with the light- and development-dependent lesion formation is identical to the behavior of *lls1* mutants in maize [33,34]. We previously reported the relationship between *lls1* and the Arabidopsis *acd1* mutant [32–34] and an orthologous mutant in rice was also reported [40]. The suppression of PaO by virus induced gene silencing in both tomato and wheat also resulted in lesion formation [37,41]. In all of these cases, a severe reduction of PaO function results in continuously expanding necrotic lesions. The expanding lesions in all of these systems only superficially resemble HR, and when examined, the underlying cellular and molecular phenotypes are distinct between PaO and HR induced lesions. Thus far, no evidence has been found to connect the accumulation of these phototoxic metabolites in disease signaling, though it remains formally possible that modulation of PaO contributes to an as yet undetermined plant-pathogen interaction.

The *lls1* gene encodes a pheophorbide a oxygenase (PaO), a key enzyme of the chlorophyll degradation pathway activated during senescence. PaO activity has thus far been reported to be restricted to senescing tissues but our results demonstrate induction by wounding in mature yet non-senescing tissues. The lack of production of superoxide and H_2_O_2_ in and around *ded1-1* lesions is consistent with *ded1* also encoding a PaO. In the absence of PaO activity, the intermediate of chlorophyll degradation that is likely to accumulate is pheide a. This molecule, a potent photosensitizer, is expected to produce singlet oxygen (^1^O_2_) upon illumination, not H_2_O_2_ and superoxide.

Singlet oxygen can kill Arabidopsis cells in a genetically-controlled process in which H_2_O_2_ and superoxide play no role [51]. Singlet oxygen causes cell death in Arabidopsis, when the PaO encoded by *acd1* is defective, by a process that is independent of superoxide and H_2_O_2_ [32]. Accordingly, *ded1/lls1/acd1* cell death likely results from direct oxidation of membrane lipids and proteins in the chloroplast by the presence of excessive ^1^O_2_. This interpretation is supported by the fact that loss of integrity of chloroplasts is the first sign of distress in cells undergoing cell death in *ded1* leaves. Indeed a close examination of the electron micrographs obtained in our study reveals that one of the first signs of chloroplast stress is the disorganization and loosening of the organization of the membranes harboring the light harvesting complexes. It is now known that at least 5 chlorophyll catabolic enzymes including PAO interact at light-harvesting complex II for chlorophyll detoxification during leaf senescence [52].

In this context, we can now begin to understand how loss of PAO within the chlorophyll catabolic complex leads to cell death. It is known that some *fluorescent* (*flu*) mutants of Arabidopsis accumulate high levels of ^1^O_2_ when exposed to light intensities that cause oxidative damage [51]. This might be the case in *lls1*/*ded1* mutants, in the absence of PaO. PaO is a key enzyme of chlorophyll catabolism and high levels of PAO within the larger catabolic complex are required during senescence to prevent accumulation of dangerous chlorophyll catabolites. Accumulation of the highly photoreactive catabolic intermediate, pheophorbide a, results in the light-dependent production of excessive ^1^O_2_ in chloroplasts. Anti-oxidative mechanisms within the chloroplast are apparently overwhelmed allowing damage of the chloroplast contents and thus initiating their destruction from within, and subsequently the death of the cell.

While the invocation of singlet oxygen may explain how *ded1/lls1* cells die, it poses a dilemma with regard to the propagation of *ded1* lesions. Singlet oxygen has such a short life (less than 100 ns) and ability to diffuse (less than 100 nm) that it is unlikely to escape *ded1* chloroplasts. While this behavior of singlet oxygen is consistent with chloroplasts being the organelle mediating cell death, it will prevent this ROS from signaling cell death propagation. Furthermore, the lack of evident H_2_O_2_ or superoxide accumulation suggests these signals cannot be responsible for lesion formation in *ded1*. The demonstration that ethephon could trigger lesions in *ded1* (Fig 6J) and *lls1*, similar to what was found for *acd1* previously [5], indicates that ethylene promotes the propagation of PaO lesions. This likely results from increased chlorophyll catabolism in response to ethylene signal transduction. It is clear from this study and others that both physical wounding and pathogen infection (but not ABA application or osmotic/cold stress) cause increased expression of the *Ded1* gene in leaves. It is widely acknowledged that ethylene, ABA, jasmonic acid (JA), and salicylic acid (SA) are positive regulators of leaf senescence and ripening. Wounding is well known to induce JA which in turn is also linked to the activation of ethylene biosynthetic genes [53]. It has been shown that JA promotes degreening via MYC2/3/4 bHLH proteins that directly bind to the Arabidopsis *PAO* promoter [54]. The suppression of *ded1* and *lls1* lesions on young leaves and activity of PaO linked to senescence also lends credence to this hypothesis. Tests in Arabidopsis seem the best way to proceed as generating double mutants of *acd1* with those that are defective either in the production or perception of ethylene should be straightforward as both are readily available.

## Material and methods

### Plant materials

The *ded1* mutant was generated by mutagenizing sorghum line P898012 with ethyl methane sulfonate (EMS) and was kindly provided by late Dr. John Axtell (Purdue University, West Lafayette, IN). Plants were grown in the field at the Agronomy Research Center at Purdue University or in pots using standard potting mix in the greenhouse at Purdue as described previously [33]. Plant phenotypes were stable across multiple years including the 1997–1999, 2002, and 2003 field seasons.

### Electron microscopy

The fourth leaf of three-week-old wild-type and *ded1* plants were wounded via pin pricking multiple times on one side of the mid-rib. At 21 and 42h post-wounding leaf tissue was harvested from the area surrounding, and including, the wound. Uninjured, control tissue was excised on the opposite side of the mid-rib of the injured leaf. Four wild-type and four mutant plants were examined at both time points. Tissue was fixed in 2.5% glutaraldehyde (v/v) in 100 mM sodium cacodylate buffer, pH 6.9 for 2.5h at 4°C. Tissue samples were post-fixed for 2h in 1% OsO_4_, 100 mM sodium cacodylate buffer, pH 6.9. The tissues were stained *en block* in 2% aqueous uranyl acetate for 1h, washed in deionized, distilled H_2_O and dehydrated through an ethanol series. The blocks were gradually infiltrated and embedded in 100% Spurr epoxy resin and polymerized at 60°C for 24h. Ultrathin sections were prepared using a diamond knife on a LKB 8800 microtome, and subsequently stained with uranyl acetate and lead citrate. The stained sections were examined on a JEOL JEM-1200EX transmission electron microscope. Images were recorded on Kodak 4489 film.

### Cloning and sequencing of *ded1-1* and its wild-type progenitor

Oligonucleotides complementary to exon 5 (GSP5 5' ACTTTTTCCAGTTCACAATGCC 3') and exon 7 (GSP8 5' GGTAGGCTGGGAGCGACAGTA 3') of the maize *lls1* gene [33] were used to amplify an approximately 1.1-kb fragment from the sorghum *ded1* mutant and its progenitor stock. These PCR products were cloned by using a TA cloning kit (Invitrogen, Carlsbad, CA) and sequenced. The complete amplification, cloning and sequencing process was completed twice for the mutant and the progenitor alleles to enable us to distinguish true sequence polymorphisms from replication errors during amplification.

### Sorghum *ded1* expression

To determine whether the expression of the *ded1* gene is altered after cellular injury, leaf number 6 of five-week-old wild-type plants was wounded by gently rubbing carborundum powder along the length of the leaf. At 1, 2, 4, 6, 8, 10 and 12h post wounding, tissue was frozen and stored at -80°C until the time of RNA isolation. Control tissue was taken at 0h. Unwounded leaves were also taken from age-matched, sibling *ded1* mutants, which displayed lesions. RNA isolation and RNA blot hybridization analysis with the ^32^P-labeled 1.1-kb sorghum *ded1* PCR amplification product described above was carried out as previously described [33].

### Linkage analysis of *ded1* mutants

DNA was isolated from two-week-old maize or sorghum seedlings by the method of Dellaporta [55] and digested with appropriate restriction endonucleases. Following gel electrophoresis, DNA blot hybridization of the digested DNA was carried out as previously described [56]. Resulting blots were probed with the ^32^P-labeled 1.1-kb sorghum *ded1* PCR amplification product described above, or with the inserts from the cloned RFLP probes *PIO000603*, *PIO000689*, *PIO000640*, *PIO000044* provided by Pioneer Hi-Bred International Inc. and *UMC157* acquired from the University of Missouri-Columbia’s Maize Mapping Center.

### Detection of H_2_O_2_ and superoxides

The H_2_O_2_ presence in lesions was detected using the DAB uptake method as described by Thordal-Christensen et al. [50]. The leaves showing lesions were cut and placed in solution containing 1mg/ml 3,3’ diaminobenzidine (DAB-HCl) pH 3.8 and incubated at room temperature for 1 hour. The leaves were then boiled in 96% ethanol to remove chlorophyll for better visualization of coloration. The presence of H_2_O_2_ was visualized as reddish brown coloration. Superoxide was detected by nitroblue tetrazolium (NBT) staining. The NBT (1mg/ml) was dissolved in 10mM NaN_3_, 10mM phosphate buffer (pH 7.8). After incubation at room temperature for one hour, the leaves were cleared of chlorophyll by boiling in 96% ethanol.
